# Risk Factors Associated With Negative Appendicectomy Rates: A Retrospective Cohort Study

**DOI:** 10.7759/cureus.64509

**Published:** 2024-07-14

**Authors:** Neil Donald, Laura Halliday, Gillian Smith, Shwetal Dighe

**Affiliations:** 1 General Surgery, Dartford and Gravesham NHS Trust, Dartford, GBR; 2 Surgery and Cancer, Imperial College London, London, GBR

**Keywords:** emergency appendicectomy, negative appendicectomy, preoperative imaging, ultrasound (us), computed tomography (ct), negative appendicectomy rate (nar), appendicitis

## Abstract

Background: Acute appendicitis (AA) is the most common emergency general surgical condition worldwide. Diagnosis is challenging and incorporates clinical, biochemical and radiological investigations. Our aim was to provide data from routine practice investigating widely utilised diagnostic methods from a single centre within the United Kingdom.

Methods: We conducted a retrospective observational cohort study of patients who underwent a laparoscopic appendicectomy for AA between April 2022 and March 2023. AA was defined as the presence of transmural polymorphonuclear leukocytes in histology. Subgroup analysis was performed on paediatric patients. Factors associated with AA were investigated, and the diagnostic utility of biochemical and radiological investigations was examined.

Results: A total of 330 appendicectomies were analysed. We found an overall negative appendicectomy rate (NAR) of 38% and 48% in paediatric patients. Independent factors associated with AA on the multivariate analysis included elevated neutrophil counts (>7 × 10^9^/L) (OR 4.04), elevated CRP (>5 mg/L) (OR 3.04) and a radiological diagnosis (OR 8.0). Computerised tomography (CT) and ultrasound had sensitivity/specificity of 98%/47% and 35%/86%, respectively. The positive-predictive values were 85% for CT and 50% for ultrasound, and the negative-predictive values were 86% for CT and 77% for ultrasound.

Conclusion: Our study has highlighted the importance of utilising a combination of factors to improve the diagnostic certainty of AA. However, our routine practice data have shown different sensitivities and specificities of imaging in comparison to existing literature, resulting in a high NAR. Further real-world data are needed to understand whether these differences from the existing data are seen in other clinical settings.

## Introduction

Acute appendicitis (AA) is the most common abdominal surgical condition in the world, with over 300,000 operations performed annually in the United States and 50,000 in the United Kingdom [1,2]. A systematic review in 2017 of 120 studies found that the pooled incidence of appendicitis range was 100 per 100,000 person-years in Northern America to 151 in Western Europe [3]. Further reports by the World Society of Emergency Surgery in 2020 and the European Association of Endoscopic Surgery (EAES) have suggested rates of 5.7-50 per 100,000 and 5.7-57 per 100,000 inhabitants, respectively [4,5]. It has further been suggested that there is a geographical difference in the lifetime risk of developing AA of 9% in the USA, 8% in Europe and 2% in Africa [6].

The diagnosis of AA is often challenging, and numerous scoring systems have been created to aid in the diagnosis. The most frequently used are the Alvarado, Appendicitis Inflammatory Response and Adult Appendicitis scores and specifically in paediatric patients the Paediatric Appendicitis Score; each system has its own unique advantages and disadvantages, and there is as yet no consensus on the most appropriate [4,7,8]. Many of these scoring systems include biomarkers but their role remains controversial, as highlighted by the EAES consensus statement in 2015. More recently, in 2020, the WSES concluded that whilst biomarkers represented a promising and reliable diagnostic tool, further evidence was still required [4,5].

It has been suggested that in the paediatric cohort white blood cell (WBC), absolute neutrophil count and C-reactive protein (CRP) are useful in predicting the presence of AA. Whilst there is an ongoing debate surrounding biomarker use, a systematic review of 58 studies suggested that AA could be ruled out if WCC, CRP and polymorphonuclear leucocyte levels are all normal [9]. However, individual markers alone lack the accuracy to predict AA [9,10].

Due to the uncertainty with clinical and biochemical indicators, imaging methods are commonly deployed to help reduce diagnostic ambiguity. A Cochrane review in 2019 found that computerised tomography (CT) has a high sensitivity and specificity, both within the 90th percentile, for diagnosing AA, and these findings were mirrored in a further meta-analysis of 37 studies in 2022 [11,12]. Ultrasound is another commonly used investigation, especially in paediatric patients and young women. However, the sensitivity and specificity are both lower than CT and the investigation is operator-dependent [12,13].

There are risks associated with a late diagnosis of AA and perforated appendicitis has a significant morbidity [14]. Paediatric patients are at an increased risk of perforated appendicitis with being younger than five commonly associated with higher perforation rates [15]. However, negative appendicectomies can also lead to complications, and surgical complications have been reported to be similar in patients undergoing a laparoscopic appendicectomy whether or not they have AA [16,17].

Negative appendicectomy rates (NARs) vary widely. A systematic review and meta-analysis of 76 thousand patients reported a wide variability of 0-46%, with a 13% NAR on meta-analysis [18]. There has been a trend of declining negative appendicectomy rates potentially due to better diagnostics, and rates of negative appendicectomies are widely reported to be under 10% with the use of diagnostic imaging and biochemical markers [19-22]. Mandatory imaging has led to reported rates of 2-3% [23,24].

The aim of our study was to evaluate diagnostic methods that are widely accessible and utilised in clinical practice within a district general hospital to determine their effectiveness individually and in combination to enhance the diagnosis of AA.

This article was previously posted to the Research Square preprint server on May 3, 2024.

## Materials and methods

We conducted a retrospective observational analysis of all patients undergoing an appendicectomy for AA over a one-year period from April 2022 to March 2023 at Dartford and Gravesham NHS Trust, a district general hospital in Dartford, United Kingdom. A total of 381 patients were initially identified. Appendicectomies performed as part of other abdominal surgery, elective or interval appendicectomies and appendicectomies performed for any reason other than AA were excluded. Fifty-one were excluded from the analysis, which resulted in 330 suitable for inclusion. Following exclusion, we recorded data regarding operative details, length of stay, biochemical markers, pathology results and imaging results from hospital electronic medical records. Data were stored following collection on Microsoft Excel (Microsoft Corporation, USA).

The definition of AA was standardised and defined as the presence of transmural polymorphonuclear leukocytes in histology. Paediatric patients were defined as those patients under the age of 16 years old at the time of presentation.

The decision to whether to perform any imaging and the selection of imaging modality was made clinically by the doctor treating the patient. The definition of positive imaging was one where the report gave a diagnosis of appendicitis. If imaging results were indeterminate, the appendix was not visualised or not commented upon; this was recorded as a negative scan result. Three patients within our cohort underwent an MRI scan, which was too small for analysis and therefore excluded from imaging results. 

Ethics guidance was not required for this study, which was defined as a clinical audit, in accordance with the NHS Health Research Authority guidance. All information within this study was collected and approved following approval and governance checks by the Dartford and Gravesham NHS Trust clinical audit department.

Statistical analysis was performed using IBM SPSS Statistics for Windows, Version 28.0 (released 2021, IBM Corp., Armonk, NY). The normality of data was assessed visually and using the Kolmogorov-Smirnov (with Lilliefors correction) and Shapiro-Wilk normality tests. Depending on their distribution, continuous variables are presented as either mean ± standard deviation or median (interquartile range, IQR). Comparison of continuous variables between those with AA and those with a negative appendicectomy was performed using the independent-samples T-test or Mann-Whitney U test, respectively. Categorical variables were compared using the chi-squared or Fisher’s exact tests. Binary logistic regression was used to determine the factors associated with AA. Two-tailed tests were used throughout with a significance level of P < 0.05.

## Results

A total of 330 patients were included in the analysis; full datasets were available for all 330 participants. A full breakdown of the demographics and admission biochemistry is shown in Table 1.

**Table 1:** Demographic breakdown and admission biochemistry. Results are displayed as medians (IQR) unless otherwise stated. ASA: American Society of Anaesthesiologists, WBC: white blood cells, CRP: C-reactive protein, ALT: alanine transaminase

	Histological diagnosis of appendicitis (n = 204)	No evidence of acute appendicitis (n = 126)	p-value
Age	32 (18-47)	23 (14-34)	<0.001
Gender, male n (%)	93 (46%)	36 (29%)	0.003
ASA Grade 1	81	53	
2	110	64	0.71
3	13	7	
4	0	2	
WBC (× 10^9^/L)	12.6 (10.1-15.7)	8.4 (6.9-11.8)	<0.001
Neutrophils (× 10^9^/L)	10.1 (7.1-12.7)	5.6 (3.7-8.2)	<0.001
Lymphocytes (× 10^9^/L)	1.4 (1.0-2.0)	1.9 (1.4-2.4)	<0.001
CRP (mg/L)	39.2 (11.7-121.9)	8.1 (1.8-51.8)	<0.001
Bilirubin (umol/L)	14.1 (10.0-20.0)	9.0 (7.0-12.0)	<0.001
ALT (U/L)	17.0 (12.0-24.5)	16.0 (12.5-25.5)	0.862

A histological diagnosis of AA, defined as the presence of transmural polymorphonuclear leukocytes, was found in 204 patients. There was no histological diagnosis of AA in 126 patients, giving an overall NAR of 38%.

As shown in Table 1, patients with AA had a higher median age (32 years vs. 23 years). AA patients had higher WBC, neutrophil count, CRP and total bilirubin levels. Patients with no histological diagnosis tended to have higher lymphocyte counts. Females accounted for 71% of all negative appendicectomies.

Radiological investigations 

A total of 204 patients underwent an imaging modality (62%). Of these, 124 (38% of study population) underwent a CT scan, and 82 (25% of study population) underwent an ultrasound scan (USS); the breakdown of these are shown in Table 2.

**Table 2:** Radiological breakdown of patients with and without acute appendicitis. CT: computerised tomography

	Histological diagnosis of appendicitis	No evidence of acute appendicitis	p-value
All imaging modalities			<0.001
Appendicitis	102 (86%)	25 (29%)	
No diagnosis of appendicitis	17 (14%)	60 (71%)	
Ultrasound			0.029
Appendicitis	8 (35%)	8 (14%)	
No diagnosis of appendicitis	15 (65%)	51 (86%)	
CT scan			<0.001
Appendicitis	92 (98%)	16 (53%)	
No diagnosis of appendicitis	2 (2%)	14 (47%)	

With all imaging modalities combined together, the sensitivity was 86% with a specificity of 71%. The positive predictive value (PPV) and negative predictive value (NPV) were 80% and 78%, respectively.

There were, however, differences within imaging modalities. In this study, CT had a sensitivity of 98% and a specificity of 47%, whilst USS had a sensitivity of 35% and a specificity of 86%. PPV values were 85% and 50% for CT and USS, respectively. The NPV values were 86% for CT and 77% for USS.

Factors predicting the diagnosis of AA

On multivariate analysis, patients with AA were four times more likely to have an elevated neutrophil count above 7 × 109/L on admission and three times more likely to have an elevated CRP above 5 mg/L on admission compared to patients who did not have appendicitis (Table 3). A radiological diagnosis of AA by either modality had an odds ratio of 8.0 for a histological diagnosis of AA (P < 0.001).

**Table 3:** Multivariate analysis of admission blood test parameters and imaging and their associations with acute appendicitis. WBC: white blood cell, CRP: C-reactive protein, ALT: alanine transaminase

Variable	Odds ratio (95% CI)	SE	p-value
WCC >11 × 10^9^/L	0.96 (0.25 to 3.61)	0.68	0.943
Neutrophil >7 × 10^9^/L	4.04 (1.11 to 14.73)	0.66	0.035
Lymphocyte >4 × 10^9^/L	0.95 (0.07 to 13.72)	1.36	0.968
CRP >5 mg/L	3.04 (1.19 to 7.78)	0.48	0.020
Bilirubin >21 umol/L	2.87 (0.70 to 11.71)	0.72	0.142
ALT >50 U/L	0.86 (0.18 to 4.16)	0.80	0.853
Radiological diagnosis of appendicitis	8.0 (3.74 to 16.90)	0.39	<0.001

A combination of WCC >11 × 109/L, neutrophil count >7× 109/L, evidence of left shift (>75% neutrophils:lymphocytes) and a CRP >5 mg/L was significant for a diagnosis of appendicitis (P < 0.001) with a specificity of 88% for the diagnosis of AA, with a positive predictive value of 80%.

Paediatric patients 

Eighty-seven paediatric patients were identified; 45 patients in this cohort had histological evidence of AA, leading to a NAR of 48%.

On subgroup analysis, there were significant differences in the total WBC, neutrophil count, CRP and total bilirubin (Table 4). There was also a significant difference in the alanine transaminase though the difference was minimal at 2.5 U/L. Forty patients underwent USS. In this population, USS could not significantly predict the presence or absence of AA (p=0.439). Both sensitivity and PPV were 36%, and specificity and NPV were 76%.

**Table 4:** Breakdown of demographic and admission biochemistry in paediatric patients. Results are displayed as median (IQR) unless otherwise stated. WBC: white blood cell, CRP: C-reactive protein, ALT: alanine transaminase

	Histological diagnosis of appendicitis (n = 45)	No evidence of acute appendicitis (n = 42)	p-value
Age	12 (8-14)	13 (11-14)	0.065
Gender, male n	17 (38%)	24 (57%)	0.071
WBC (× 10^9^/L)	13.9 (9.5-16.1)	8.0 (6.7-10.3)	<0.001
Neutrophils (× 10^9^/L)	11.5 (6.7-13.5)	4.9 (3.3-7.0)	<0.001
Lymphocytes (× 10^9^/L)	1.2 (0.9-2.2)	2.1 (1.4-2.4)	0.001
CRP (mg/L)	21.6 (7.4-118.7)	3.5 (0.5-25.5)	<0.001
Bilirubin (umol/L)	12.0 (9.0-14.0)	8.0 (6.8-12.0)	0.002
ALT (U/L)	12.0 (10.0-16.0)	14.5 (12.0-18.8)	0.025

## Discussion

In this single-centre study within the United Kingdom, we determined a negative appendicectomy rate of 38% based on strict histological criteria, which is slightly higher than reported literature in the field [9,18-23]. Our results though should be interpreted in the context of requiring strict histological criteria of transmural polymorphonuclear leukocyte infiltration, rather than the wider criteria of any inflammation. In addition, the inter-institution variability of routine use of laparoscopy as a diagnostic tool to aid in the diagnosis whilst proceeding to an appendicectomy concurrently during the same procedure increases negative appendicectomy rates and similar rates have been published [24,25].

In our cohort, we found that females were more at risk of undergoing a negative appendicectomy. This may arise due to other gynaecological conditions that may present with similar signs and symptoms and is in keeping with the known literature at similar rates [9,26-29]. Moreover, in keeping with other literature, we found advancing age to be negatively associated with a negative appendicectomy [8,19,21,26,27]. This may be due to older patients being more likely to undergo cross-sectional radiological imaging to exclude other diagnoses that can mimic AA or identify any underlying pathology that may cause AA in older patients [4].

The findings in this study cohort have suggested USS is of no discernible benefit in paediatric patients. However, less than half of the paediatric patients in this study underwent ultrasound and the sample size may be too small to detect significant differences. Further research is needed to examine the local practices around the decision to request pre-operative imaging in children in this study population. 

The sensitivity of USS in this study was very low, with only 35% of AA patients who had a USS having a positive finding of AA on scanning, indicating that it is of little use in excluding AA. In addition, the PPV value of 50% gives little value in confirming a histological diagnosis of AA. By contrast, CT imaging had 98% sensitivity and was very good in successfully excluding AA. This clear advantage of CT imaging in the diagnosis of AA has to be weighed against the associated radiation exposure and low specificity in this study, with 53% of cases reported as AA resulting in a negative appendicectomy.

However, as previously discussed, other studies have shown higher specificities and sensitivities for preoperative imaging, and therefore further research is needed to understand the reasons behind the lower specificities and sensitivities in this study population [11-13,16].

Whilst we did find a significant difference in the total WBC between those who did and did not have AA, we did not find on multivariate analysis a WBC >11,000 independently increasing the odds of an AA diagnosis. However, this is in contrast to some of the existing literature [9,21,28,29]. Other studies have suggested that WBC is not a predictor of AA [30]. Furthermore, it has been suggested that WBC is a poor predictor of AA due to unreliable specificity and sensitivity, and there is no consensus for a reliable cut-off value [31]. Potential reasons for this are other inflammatory or infectious conditions that can mimic appendicitis, such as mesenteric lymphadenitis, gastroenteritis, urinary tract infections or tube-ovarian pathology. We did however find that patients with AA were four times more likely to have an elevated neutrophil count at admission in comparison to those with a negative appendicectomy, which is in keeping with other literature [31,32].

With the increasing use of imaging as a key diagnostic tool, this study highlights important limitations in its use for the diagnosis of AA and the need to continue pursuing a combination approach that includes clinical scoring systems, multiple biomarkers and the use of radiological imaging. Further research is needed to implement a new scoring system, taking into account a combination approach of clinical signs and symptoms together with biomarkers in patients who may be unable to undergo imaging modalities and where there is diagnostic uncertainty. We also suggest further research from various centres using real-time practice data to further investigate the utility of imaging to either exclude or confirm the diagnosis of AA and its role in influencing local NAR.

Limitations

A limitation of our study was that despite the high number of patients, it is a single-centre study. There is furthermore the potential for inter-operator variability in interpreting imaging, which may affect results. Data were not reliably recorded on clinical features or perimeters, such as heart rate, temperature or examination findings, and therefore could not be included in these analyses.

## Conclusions

We have demonstrated in our cohort that an elevated neutrophil, elevated CRP count and a radiological diagnosis are independent risk factors on multivariate analysis for a diagnosis of AA. Our findings have also demonstrated that the use of several biochemical markers in combination can increase the confidence with which a diagnosis can be made. The usefulness of CT imaging to reduce the likelihood of missed AA has been highlighted. However, we found a low specificity, and therefore it may be of limited use in reducing a high NAR in clinical practice. USS was of less diagnostic utility, even in paediatric groups. Within our routine practice, there was discordance with radiological sensitivity and specificity values compared with those described in prior studies.
